# Editorial: Paleoradiology and mummy studies for disease identification

**DOI:** 10.3389/fmed.2026.1783130

**Published:** 2026-02-03

**Authors:** Sahar N. Saleem, Dario Piombino-Mascali

**Affiliations:** 1 Department of Radiology, Kasr Al Ainy Faculty of Medicine, Cairo University, Cairo, Egypt; 2 Department of Anatomy, Histology, and Anthropology, Faculty of Medicine, Vilnius University, Vilnius, Lithuania

**Keywords:** ancient diseases, archaeology, computed tomography, mummy, paleoradiology, X-ray

Paleoradiology uses modern imaging techniques to study the remains of ancient individuals, including mummies and other preserved materials. The field has fundamentally transformed our approach to ancient civilizations. By applying methods such as computed tomography (CT) and digital radiography to human remains, researchers can now non-invasively investigate the lives, health, and deaths of past populations—moving beyond speculation to produce rigorous scientific data.

This Research Topic, “*Paleoradiology and Mummy Studies for Disease Identification*,” curated by editors Sahar Saleem and Dario Piombino-Mascali, showcases the remarkable capabilities of this evolving discipline. The collected studies demonstrate paleoradiology's power not merely as a diagnostic tool but as an integral component of archaeometry—a multidisciplinary field combining imaging science with chemistry, microscopy, and anthropology to replace historical myths with empirical evidence and reshape our understanding of ancient societies (Figure 1).

**Figure 1:**
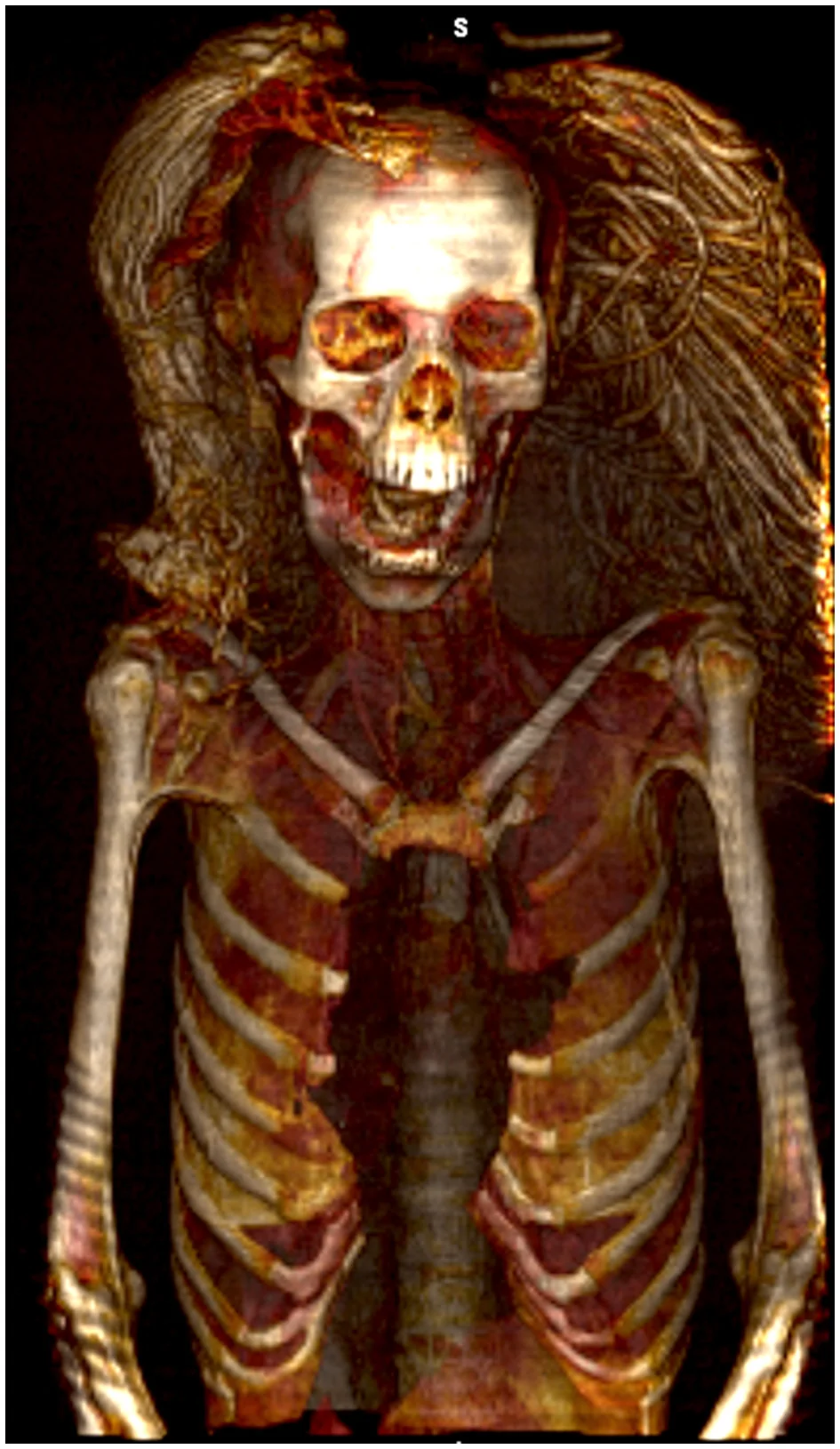
Colored 3D CT reconstruction (frontal view) of a mummified ancient Egyptian woman (ca. 1479-1458 BC). This individual, known as the ‘Screaming Woman,’ shows a widely open mouth and a detailed wig. The non-invasive model integrates skeletal structures, including rib-cage detail, preserved soft tissues, and the texture of a thick, wavy wig. © Saleem SN. The identity matches the individual described as the ‘Screaming Mummy’ in Saleem and El-Merghani.

To appreciate the contributions of these studies, we review them in chronological order, tracing a path of scientific discovery from the Bronze Age to the Modern Era.

Our journey begins with the earliest case: the identification of leprosy in Bronze Age Oman (2500–2000 BCE). The study by Robbins Schug et al. addresses the challenge of analyzing fragmentary and commingled skeletal remains. Using micro-CT scanning, the authors characterized the natural history of leprosy progression at an ultrastructural level in three archaeological maxillae. Their analysis revealed features consistent with lepromatous leprosy, including atrophy of the anterior nasal spine, resorption of the medial alveolar process, deterioration of the piriform aperture margin, and atrophy of the nasal septum. This finding provides crucial evidence for early migrations of *Mycobacterium leprae* and demonstrates the utility of micro-CT in non-invasively characterizing pathology in isolated skeletal elements. The results offer new insights into health and disease in Bronze Age Arabia, illuminating the environmental and social factors that shaped disease patterns.

Moving forward to Egypt's 18th Dynasty (ca. 1479–1458 BCE), the study of “The Screaming Mummy” by Saleem and El-Merghani revolutionizes our understanding of mummification practices. Combining CT with Scanning Electron Microscopy, Fourier-Transform Infrared Spectroscopy, and X-ray Diffraction Analysis, the researchers analyzed skin, hair, and wig samples. CT imaging revealed the mummy's morphology, estimated age at death, and the presence of retained viscera, indicating a non-evisceration mummification method. Advanced analytical tests identified costly imported materials—juniper resin, frankincense, and henna—used externally to preserve the body. This finding challenges the long-held belief that retained viscera reflected poor mummification. Instead, the study demonstrates that external application of high-quality resins was sufficient for effective preservation. The research also addresses the mummy's open-mouthed “screaming” appearance, suggesting it may have resulted from cadaveric spasm rather than embalming error. Overall, this work transforms our understanding of mummification quality and trade in embalming materials during the 18th Dynasty.

Progressing to 17th−18th century Lithuania, the work of Piombino-Mascali et al. on the Kedainiai elite exemplifies the value of integrated approaches. The team examined eight high-ranking individuals using classical anthropological methods and CT to assess biological indicators, pathology, and preservation. Findings included post-mortem manipulation and various pathological changes—degenerative joint disease, lung and arterial calcifications, and neoplasms—that would have been undetectable without paleoradiology. The study underscores the need for continuous monitoring of mummified remains, particularly given observable decay over recent decades, and demonstrates how combining paleopathology with imaging generates lasting datasets that support interpretation even when the remains themselves face environmental or political threats.

From the same period in 17th−19th century Italy, Larentis, Gorini, Campus, Lorenzetti et al. studied three hermit mummies from the Sanctuary of Madonna della Corona, close to the border between Veneto and Trentino-Alto Adige. Due to the sanctuary's remote cliffside location (775 m elevation), the researchers used lightweight, portable X-ray equipment for radiological examination. This innovative approach highlights the potential of next-generation imaging for studying clothing, restoration history, taphonomy, and biological remains in otherwise inaccessible contexts. Their interdisciplinary analysis revealed new historical and biological data about these devotional mummies, previously unknown to both Italian and international scholars, demonstrating that mobile radiography can be the optimal tool when conventional CT is not feasible.

In late modern Palermo, Sicily (1787–1880 CE), Squires et al. examined 41 mummified non-adults from the world-renowned Capuchin Catacombs. Using portable X-ray imaging, they explored demographics, health, possible causes of death, and funerary treatment. The study revealed that mortuary rites were primarily influenced by family wealth and social status rather than age or health at death. The absence of metabolic, neoplastic, or traumatic lesions suggests deaths were mostly due to acute illnesses. Even children with chronic conditions were afforded similar rites. Artifacts associated with mummification and display were also documented, illustrating how non-invasive imaging provides comprehensive insight into the lives and deaths of non-adults at the time.

To conclude this journey through time, Larentis, Gorini, Campus, Vanin et al. present the study of six non-adult petrified specimens prepared by the Italian scientist Paolo Gorini (1813–1881) in Lodi, Lombardy, during the 19th century. Preserved since 1981 in the Old Hospital, these individuals constitute the only known examples of Gorini's preserved children. Using paleoradiology, the researchers documented their biological characteristics, embalming techniques, and conservation state. Radiographic analysis revealed detailed insights into skeletal and dental development and pathological conditions associated with physiological stress and metabolic bone disease. Exceptional soft-tissue preservation allowed identification of dermal, muscular, and visceral structures, while modifications such as intra-orbital inserts reflected Gorini's concern for anatomical realism. The findings demonstrate the effectiveness of Gorini's petrification technique—now better understood through rediscovered embalming formulae—which achieved both structural fidelity and long-term stability even in fragile infants. Beyond its technical success, the study provides new biomedical data on health, disease, and preservation in 19th-century non-adult populations, situating Gorini's anatomical work within the broader history of experimental anatomy and conservation science.

## Conclusion

The studies presented in this Research Topic clearly show that paleoradiology has become a cornerstone of modern archaeology. No longer a mere auxiliary tool, it stands at the center of a methodological shift toward archaeometry. The synergy of imaging science with chemistry and materials analysis is transforming the discipline from interpretive speculation into data-driven, empirical inquiry. Paleoradiology advances mummy studies by revealing disease histories, injuries, causes of death, and cultural practices.

As imaging technologies evolve—from standard radiography to high-resolution micro-CT and portable digital systems—our ability to explore the past becomes increasingly detailed. The future of archaeological research lies in integrating these advanced imaging tools with other scientific methods. Paleoradiology provides the structural framework for this evidence-based understanding, ensuring that we can continue uncovering the authentic stories of our ancestors and safeguarding our shared cultural heritage for generations to come.

